# *Frullania knightbridgei*, a new liverwort (Frullaniaceae, Marchantiophyta) species from the deep south of Aotearoa-New Zealand based on an integrated evidence-based approach

**DOI:** 10.3897/phytokeys.8.2496

**Published:** 2012-01-02

**Authors:** Matt von Konrat, Peter de Lange, Matt Greif, Lynika Strozier, Jörn Hentschel, Jochen Heinrichs

**Affiliations:** 1 Department of Botany, The Field Museum, 1400 South Lake Shore Drive, Chicago IL 60605-2496, USA; 2 Ecosystems and Species Unit, Department of Conservation, New Zealand; 3 Biology Department, Wilbur Wright College, 4300 N. Narragansett, Chicago, IL, USA; 4 Department of Systematic Botany with Herbarium Haussknecht and Botanical Garden, Friedrich Schiller University, Fürstengraben 1, 07743 Jena, Germany; 5 Department of Systematic Botany, Albrecht von Haller Institute of Plant Sciences, Georg August University, Untere Karspüle 2, 37073 Göttingen, Germany

**Keywords:** Frullaniaceae, *Frullania*, subg. *Microfrullania*, *Frullania knightbridgei* sp. nov., morphology, DNA sequence data, New Zealand Flora, halotolerant liverwort

## Abstract

*Frullania* is a large and taxonomically complex genus. A new liverwort species, *Frullania knightbridgei* **sp. nov.** from southern New Zealand, is described and illustrated. The new species, and its placement in *Frullania* subg. *Microfrullania*, is based on an integrated evidence-based approach derived from morphology, ecology, experimental growth studies of plasticity, as well as sequence data. Diagnostic characters associated with the leaf and lobule cell-wall anatomy, oil bodies, and spore ultra-structure distinguish it from all other New Zealand species of *Frullania*. A critical comparison is also made between *Frullania knightbridgei* and morphologically allied species of botanical regions outside the New Zealand region and an artificial key is provided. The new species is similar to some forms of the widespread Australasian species, *Frullania rostrata*, but has unique characters associated with the lobule and oil bodies. *Frullania knightbridgei* is remarkably interesting in comparison with the majority of *Frullania* species, and indeed liverworts in general, in that it is at least partially halotolerant. Maximum parsimony and maximum likelihood analyses of nuclear ribosomal ITS2 and plastidic *trn*L-*trn*F sequences from purported related speciesconfirms its independent taxonomic status and corroborates its placement within *Frullania* subg. *Microfrullania*.

## Introduction

*Frullania* Raddi (Frullaniaceae) is a large and complex liverwort (Marchantiophyta) genus with a worldwide distribution (Yuzawa 1991, von Konrat and Braggins 2001a). The number of published *Frullania* names has been reported to be over 2000 (von Konrat et al. 2010). Published estimates of the number of accepted species for the genus that have become widely recognized range from 300–375 accepted species (e.g., Schuster 1992, Gradstein et al. 2001). However, there is no evidence whatsoever to support these suppositions as no worldwide monographic treatment of *Frullania* has ever been attempted (von Konrat et al. 2006a, 2010). On the contrary, growing data and evidence may suggest the number of 300–375 species is a minimum estimate at best (von Konrat et al. 2010); in some cases the underestimation of the *Frullania* species diversity has been attributed to conservative morphology within species complexes (Heinrichs et al. 2010). In New Zealand, the current number of accepted taxa is 33, including 31 species and two varieties of which 10 are apparently endemic (Hattori 1979a, b; 1983; Glenny 1998; von Konrat and Braggins 2005; von Konrat et al. 2006b; von Konrat et al. 2010, 2011, this issue). Here we present a study of a newly discovered species that is morphologically close to *Frullania rostrata* (Hook. f. et Taylor) Hook. f. et Taylor, which is considered a widespread, polymorphic and common Australasian species (von Konrat et al. 2006b). The new species would be lumped under *Frullania rostrata* based on overall gross morphology.

Our paper is part of a broader, on-going, regional study of *Frullania* species by us that includes the botanical regions of New Zealand, Australia, the Pacific, South East Asia, and South America. Our new species occurs on Stewart Island/Rakiura and the Auckland Islands group of the New Zealand botanical region (as defined by de Lange and Rolfe 2010). Both areas are regarded as extremely biologically and biogeographically significant (Given and Hnatiuk 1995; McGlone and Wilson 1996; Wilson 1987; Wagstaff et al. 2011). Stewart Island/Rakiura, is the southernmost and third largest island of the New Zealand archipelago, with about 85% of the island comprising Rakiura National Park (Heenan et al. 2009). Although the fauna and flora has been partially modified as a consequence of historical Maori and European settlement, the island’s vegetation patterns are little altered from their pre-human state (Wilson 1987). On the other hand, the Auckland Island group is one of five New Zealand sub-Antarctic island groups (including Snares, Bounty, Antipodes, Auckland, and Campbell islands) and are all World Heritage Areas (Chown et al. 2008). The distinctive flora of the subantarctic islands includes some of the last remnants of a once-diverse Antarctic flora, with examples of many plants possibly still retaining distinctive features of their ancestors (Wagstaff et al. 2011).

The new species of *Frullania* described below is assigned to *Frullania* subg. *Microfrullania* (R.M.Schust.) R.M.Schust., which is confirmed by molecular evidence.Detailed microscopic and SEM micrographs as well as a brief comparison with morphologically similar species are provided. This new species is remarkable in comparison with the majority of *Frullania* species in New Zealand for its least partial tolerance and exposure to salt spray. For consistency and clarity through this article, the results and discussion that follows refers to the newly recognised species as *Frullania knightbridgei*.

## Methods

### Living material

In order to monitor the stability of character-states and assess whether some characters varied independently of the genotype, material was grown under uniform environmental conditions described by von Konrat and Braggins (2001a). The same individual colonies were grown under three different light and water regimes and characters subsequently monitored for stability or variation. Voucher specimens of cultivated material are deposited at AK and F.

### Herbarium material

Dried herbarium material was examined from AK, AKU, CANB, BM, CRI, F, G, GOET, MEL, MELU, MPN, NICH, NY, P, S, WELT, and WTU. Herbarium acronyms follow Holmgren and Holmgren (2003).

### Morphological study

Where necessary, plant material was cleared to remove pigmentation using the method outlined by von Konrat and Braggins (2001a) and the cell layers of the capsule wall were separated as described by von Konrat et al. (1999). Microscopy techniques, measurements, the use of descriptors to indicate abundance and frequency, terminology of spore ornamentation, preparation of material (including spores for the SEM studies) are outlined in detail by von Konrat and Braggins (2001b) and von Konrat et al. (2006a, b).

### DNA extraction, PCR amplification and sequencing

Dried tissue was disrupted with the aid of a sterile steel bead in a Qiagen tissuelyser (Qiagen Inc.Hilden, Germany) set at 30 Hz for 45 s. Genomic DNA was extracted and purified using an Invisorb Spin Plant Mini Kit (Invitek, Berlin, Germany) according to the manufacturer’s specifications. Two molecular markers, the internal transcribed spacer 2 of nuclear ribosomal DNA, and the plastidic *trn*L-F region were sequenced using the primer sets presented by Hartmann et al. (2006) and Gradstein et al. (2006). Approximately 525 base pairs (bp) of the 5.8S-nrITS2 region were sequenced, along with 500 bp of the *trn*L-*trn*F region per isolate. PCR for each sample was performed in a total of 25 μl and contained 2.5 μl dntp mix, 2.5 μl MgCl_2_, 5 μl of Bovine Serum Albumin (New England Biolabs, Ipswich, Massachusetts, USA) 1 μl of the forward primer, 1 μl of the reverse primer, 0.5 μl Taq (Roche diagnostics, Indianapolis, Indiana, USA), 10.5 μl of dH_2_0, and 2 ul of sample DNA. PCRs were run for 37 cycles in a Dyad DNA engine (Bio-Rad Laboratories, Inc., Hercules, California, USA) set to the following parameters: initial denaturation at 94°C for 2 min, denaturation at 94°C for 1 min, annealing at 55°C for 1 min, extension at 72°C for 1 min, then final extension at 74°C for 7 min followed by a cool down stage at 4°C. The amplicon was purified using a Nucleofast 96 well PCR plate (Macherey-Nagel, Evanton, Pennsylvania, USA). Cycle sequencing was performed using the same primer sets as for the PCRs.  Sequencing reactions were done using the BigDye terminator cycle sequencing kit (Applied Biosystems, Foster City, California, USA) and amplicons were run on an ABI 3730 (Amersham Pharmacia Biotech Inc., Piscataway, New Jersey, USA). A consensus sequence was constructed and edited using Sequencher version 4.10 (Gene Codes Corp., Ann Arbor, Michigan, USA).

### Taxon sampling and outgroup selection

Initially, the new sequences were compared with GenBank sequences using the BLASTN program (Altschul et al. 1990). The BLAST searches confirmed the position within *Frullania* subg. *Microfrullania*.Ingroup taxa representing representatives of this subgenus were selected to test taxonomic hypotheses based on morphology. Several accessions of *Frullania rostrata* were included because *Frullania knightbridgei* shares several morphological characters with this morphologically rather variable taxon. Based on the analyses of Hentschel et al. (2009), three representatives of *Frullania* subg. *Thyopsiella* [*Frullania asagrayana* Mont., *Frullania microphylla* (Gottsche) Pearson, *Frullania tamarisci* (L.) Dumort.] were designated as outgroup taxa for phylogenetic reconstruction. Taxa studied, including GenBank accession numbers and voucher details, are listed in Table 1.

**Table 1.** *Frullania* taxa used in the present study, including information about the origin of the studied material, voucher information, and the herbarium where the voucher is deposited, as well as GenBank accession numbers. Sequences in bold were obtained from GenBank.

**Taxon**	**Origin**	**Voucher**	**GenBank accession number**	
			**ITS2**	***Trn*L-*trn*F**
*Frullania asagrayana*	U.S.A.	Davison 6912	**FJ380498**	**FJ380344**
*Frullania knightbridgei* von Konrat & de Lange	Stewart Island, New Zealand	von Konrat 99/12-09(AK)	JQ283996	JQ284003
*Frullania lobulata* (Hook.) Hook. & Nees	Chile	Buck 39518 (GOET)	**FJ380305**	**FJ380465**
*Frullania magellanica* F. Weber & Nees	Chile	Engel 25265 (GOET)	**FJ380464**	**FJ380304**
*Frullania microcaulis* Gola	Chile	Engel 25351 (GOET)	**FJ380466**	**FJ380306**
*Frullania microphylla* (Gottsche) Pearson	Tenerife	Eckstein 2287 (GOET)	**FJ380512**	**FJ380358**
*Frullania parhamii* R.M. Schust.	Fiji	von Konrat 6/14-27 (GOET)	**FJ380463**	**FJ380303**
*Frullania rostrata* (Hook.f. & Taylor) Hook.f. & Taylor ex Gottsche et al.	South Island (I), New Zealand	Engel & von Konrat 27369 (GOET)	**FJ380461**	**FJ380301**
*Frullania rostrata*	South Island (II), New Zealand	Schäfer-Verwimp & Verwimp 13897 (GOET)	**FJ380462**	**FJ380302**
*Frullania rostrata*	Motukowhai Island, New Zealand	Cameron 12503 (AK)	JQ283999	JQ284006
*Frullania rostrata*	Raoul Island (I), Kermadec Islands	Stanley s.n. (AK)	JQ284000	JQ284007
*Frullania rostrata*	Raoul Island (II),<br/> Kermadec Islands	de Lange & Havell K376 (AK)	JQ284001	JQ284008
*Frullania rostrata*	North Cape (I), <br/> New Zealand	de Lange 8034 (AK)	JQ283997	JQ284004
*Frullania rostrata*	North Cape (II), <br/> New Zealand	de Lange 8037 (AK)	JQ283998	JQ284005
*Frullania* sp.	North Cape, New Zealand	de Lange 8038 (AK)	JQ284002	JQ284009
*Frullania tamarisci*	Germany	Hentschel Bryo0733	**FJ380502**	**FJ380348**

### Phylogenetic analyses

All sequences were aligned manually in Bioedit version 7.0.5.2 (Hall 1999). Ambiguous positions were excluded from the alignment and lacking parts of sequences were coded as missing. Maximum parsimony (MP) and maximum likelihood (ML) analyses were carried out with PAUP* version 4.0b10 (Swofford 2000).

MP heuristic searches were conducted with the following options: heuristic search mode, 1.000 random-addition-sequence replicates, tree bisection-reconnection (TBR) branch swapping, MULTrees option on, and collapse zero-length branches off. All characters were treated as equally weighted and unordered. Non-parametric bootstrapping values (Felsenstein 1985) were generated as heuristic searches with 1.000 replicates, each with ten random-addition replicates. The number of rearrangements was restricted to ten million per replicate. Bootstrap percentage values (BP) above 70 were regarded as good support (Hillis and Bull 1993). Where more than one most parsimonious tree was found, trees were summarised in a strict consensus tree.

The two genomic regions were first analysed separately to check for incongruence. The strict consensus trees of the non-parametric bootstrap analyses were compared by eye to identify conflicting nodes supported by at least 70% (Mason-Gamer and Kellog 1996). The trees gave no evidence of incongruence. Hence the datasets were combined. jModeltest 0.1 (Posada 2008) was used to select the TIM2 + G model of evolution for the ML analysis of the combined dataset. The analysis was performed as heuristic search using ten random-sequence addition replicates, MULTrees option on, collapse zero length branches off, and TBR branch swapping. The confidence of branching was assessed with PAUP* using 200 non-parametric bootstrap resamplings generated as heuristic searches.

### Species concept

Although the determination of species is regarded as one of the most important activities of the taxonomist, the majority of systematists undertaking monographs and revisions of vascular plants do not discuss the concepts or the criteria to delimit species  (McDade 1995). A similar statement can undoubtedly be applied to liverwort systematics (von Konrat et al. 2006a, 2010). Here, we adopt a hierarchical model as promoted by Mayden (1997). This model considers the Evolutionary Species Concept as a theoretically robust primary species concept, as well as a bridging, secondary or operational species concept. This is discussed in the context of *Frullania* by von Konrat et al. (2006a).

## Data resources

The occurrence data underpinning the analysis has been uploaded as a Darwin Core Archive (DwC-A), to the Global Biodiversity Information Facility (GBIF) via the Pensoft Data Hosting Center at the GBIF’s Integrated Publishing Toolkit (IPT) (http://ipt.pensoft.net/ipt/manage/resource.do?r=deep_south_frullania_species). The genomic sequences are deposited at GenBank and their hyperlinked accession numbers are listed in Table 1.

In addition to the current paper semantically tagged and enhanced using the Pensoft Mark Up Tool (PMT), repository data and images, including images with zoom capability can also be accessed at www.discoverlife.org and www.symbiota.org for selected species that are closely allied to the newly described species. The Consortium of North American Bryophyte Herbaria (CNABH) was created to serve as a gateway to distribute data resources of interest to the taxonomic and environmental research community, offering a common web interface, including tools to locate, access and work with a variety of data (see http://symbiota.org/bryophytes/index.php).

*Frullania rostrata* can be accessed at the following urls:

http://www.discoverlife.org/mp/20q?search=Frullania+rostrata

http://symbiota.org/bryophytes/taxa/index.php?taxon=Frullania%20rostrata

http://symbiota.org/bryophytes/taxa/index.php?taxon=166175

http://emuweb.fieldmuseum.org/botany/botanytaxDisplay.php?irn=93741

*Frullania magellanica* can be accessed at the following url’s:

http://www.discoverlife.org/mp/20q?search=Frullania+magellanica

http://symbiota.org/bryophytes/taxa/index.php?taxon=166024

*Frullania truncatistyla* can be accessed at the following url:

http://symbiota.org/bryophytes/taxa/index.php?taxon=232649

## Results and discussion

In the present study, hypotheses of species differences are based on support from multiple lines of evidence, including morphology, experimental growth studies, and nucleotide sequences. This is discussed below.

### Phylogeny

**Figure 1.**
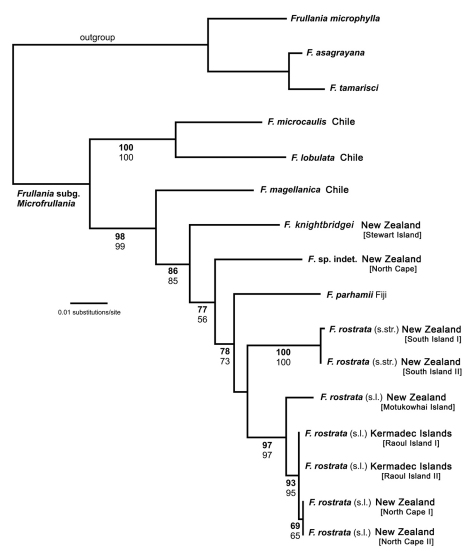
Maximum likelihood phylogeny (ln = -2984.6458) derived from an nrITS2 – *trn*L-F sequence alignment including 14 new sequences and 18 sequences from Hentschel et al. (2009). ML bootstrap percentage values (> 50) in bold face, MP bootstrap percentage values (>50) not bold.

Of a total of 964 molecular characters, 127 were parsimony informative, 62 autapomorphic, and 775 constant (Table 2). The MP analysis resulted in two trees of 304 steps with a consistency index of 0.78 and a retention index of 0.80 (not depicted). A single most likely tree was found in the ML analysis (Fig. 1, ln = -2984.6458). MP and ML topologies differ only slightly. In the MP topology, *Frullania parhamii* R.M.Schust. is placed in a polytomy with the *Frullania rostrata* (Hook.f. & Taylor) Hook.f. & Taylor ex Gottsche, Lindenb. & Neesrepresentatives whereas in the ML trees, *Frullania parhamii* is placed sister to *Frullania rostrata* in an unsupported relationship. *Frullania knightbridgei* is well separated from *Frullania rostrata*, and placed in a paraphyletic grade with other members of *Frullania* subg. *Microfrullania*. The morphologically similar *Frullania truncatistyla* von Konrat, Hentschel, Heinrichs & Braggins has not yet been included in molecular studies.

**Table 2.** Distribution of constant and phylogenetically informative sites for aligned positions of the two genomic regions.

	**L-F*trn***	**ITS1-5.8S-ITS2**	**Total**
Number of sites in matrix	516	448	964
constant	465	310	775
autapomorphic	28	34	62
parsimony informative	23	104	127

*Frullania rostrata* is split in two robust subclades. Hence, the ML phylogeny indicates that *Frullania rostrata* - despite exclusion of *Frullania knightbridgei* - is part of a species complex, possibly with some geographical structure. This may be supported by the significant number of synonyms and herbarium specimens summarised under *Frullania rostrata* (von Konrat et al. 2006a). *Frullania rostrata* might well be regarded as a Southern Hemisphere equivalent of the Holarctic *Frullania tamarisci*. Heinrichs et al. (2010) investigated *Frullania tamarisci*, which is typically regarded as a single polymorphic species. Using sequences from the nrITS region and plastid *trn*L-*trn*F and *atp*B-*rbc*L, their analyses resolved eight partly sympatric monophyletic groups representing distinct species rather than subspecies or varieties.

The number of molecular studies at the population level in liverworts is still limited. This hampers our efforts to quantify the contribution of cryptic species to the global biodiversity of liverworts (von Konrat et al. 2010). Existing studies suggest a significant part of bryophyte biodiversity is undetected with traditional morphological concepts alone (Heinrichs et al. 2009). It is clear, we urgently need more species-level phylogenies with extensive population sampling to approximate the actual diversity of *Frullania*, and to elucidate speciation processes and distribution range formation (Bombosch et al. 2010, Heinrichs et al. 2010, Ramaiya et al. 2010).

### Growth studies

In *Frullania*, as well as liverworts generally, there remains a large gap between characters used for delimitation and our understanding and knowledge of their plasticity in nature (von Konrat et al. 2006b). *Frullania rostrata* and the new species, *Frullania knightbridgei*, responded well to growing in controlled environmental conditions in a glasshouse unit. Oil bodies in particular were monitored. The stability of oil body characters indicates that the differences have some underlying genetic basis; thus it is likely that the salient characters of this species are genetically dependent rather than influenced by the environment.

### Morphology

Many critical morphological features have often been neglected in liverwort systematics (Schuster 1992; von Konrat et al. 1999, 2001a, 2006b), and scores of studies have been restricted to herbarium material where ephemeral structures; e.g., sporophytes and oil bodies, have not been available (von Konrat et al. 2006a, b; Heinrichs et al. 2009). The new species is morphologically aligned to a group of species representing *Frullania* subg. *Microfrullania*, which has been resolved as a monophyletic group (Hentschel et al. 2009). Inclusion of *Frullania knightbridgei* in *Frullania* subg. *Microfrullania* is also supported by molecular evidence as discussed above. *Frullania* subg. *Microfrullania* represents a clade with the most historical confusion out of all *Frullania* subgenera with taxa occurring in southern South America, Australasia and islands of the South Pacific, New Guinea, and Indonesia (von Konrat et al. 2006a, 2010).

**Figure 2.**
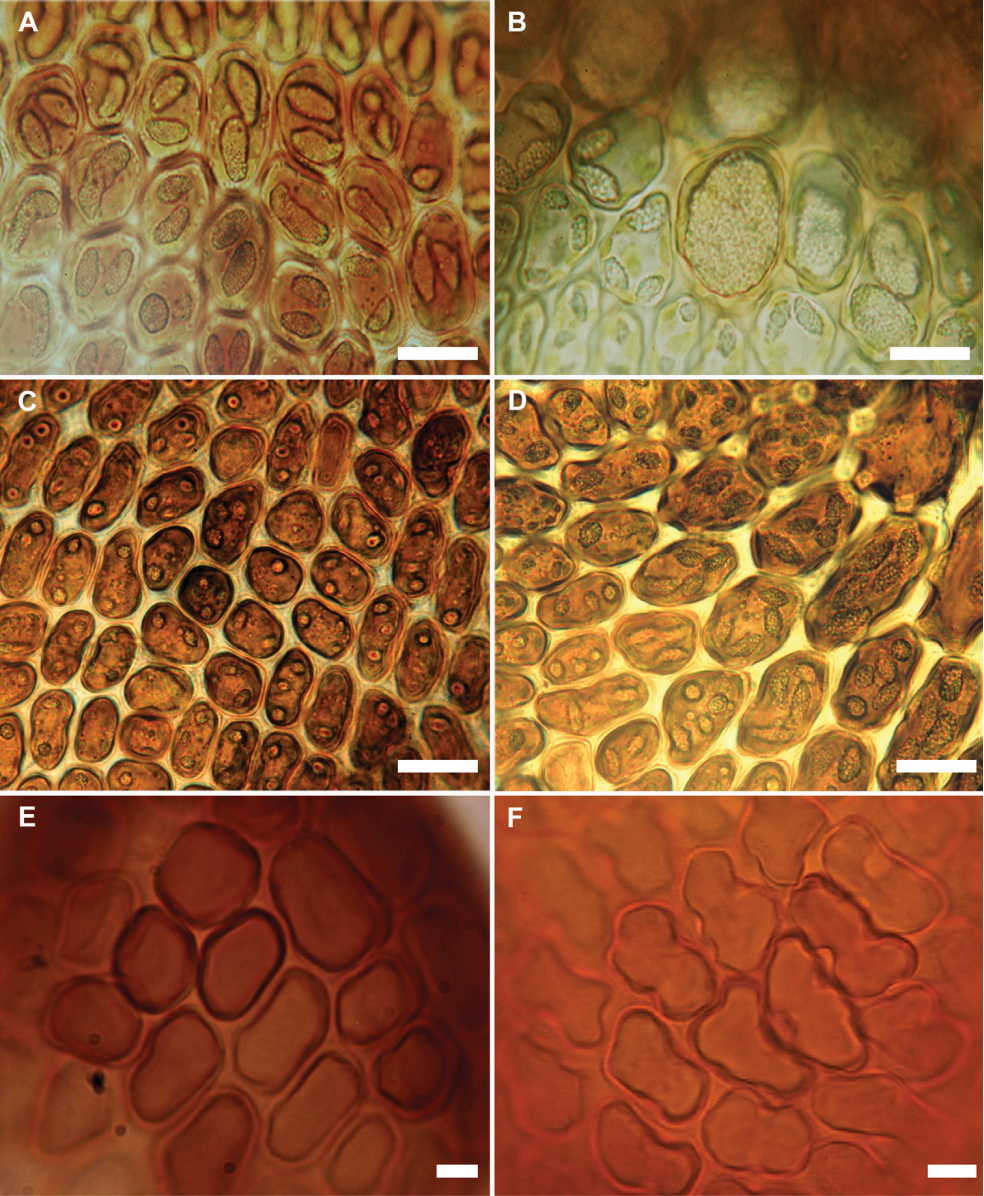
Variation in characters associated with the leaf-lobe oil bodies and leaf-lobule anatomy (**A, B, E** *Frullania knightbridgei*; **C, D, F** *Frullania rostrata*) **A** Oil bodies of the median region of the leaf-lobe, very large, (1)2–(3) per cell, collectively occupying over 75% of the cell lumen **B** Oil bodies of basal cells, a characteristic group of basal ocelli, each ocellus almost occupying the entire cell lumen **C** Oil bodies of median cells, 2–3 per cell, collectively occupying very small area of cell lumen, lacking any significant ornamentation and appearing as almost homogeneous oil droplets **D** Oil bodies of basal cells, 3–5 per cell **E** Semi straight cell walls toward apex leaf-lobule **F** Flexuose cell walls towards apex of leaf lobule. Scale bars A, B = 15 µm; C–F = 10 µm.

The new species appears almost to lie intermediate between *Frullania rostrata*,of New Zealand and Australia, and *Frullania pseudomeyeniana* S. Hatt. of New Caledonia. The latter is only known from the type material (New Caledonia, Mont Mou, N of Paita, 1100 m., *Kitagawa 21422*, NICH), which was examined by the senior author. *Frullania knightbridgei* also has some similarity with *Frullania magellanica* (Spreng.) F. Weber et Neesof Chile. *Frullania knightbridgei* superficially strongly resembles some forms of *Frullania rostrata* in plant size, the large styli and lobules, and the entire underleaves. However, with fresh material, *Frullania knightbridgei* is immediately discernable from *Frullania rostrata* by the presence of large oil bodies (usually only 2 per cell, occasionally 1 or 3) that almost occupy the entire cell lumen of basal and median cells of the leaf lobe (Fig. 2a–b). In the absence of oil body data, careful consideration has to be given to the anatomy of the leaf-lobe and -lobule to help differentiate between these species. In *Frullania knightbridgei*, cells towards the lobule apex progressively develop a more regular shape (quadrate to rectangular) and the cell walls become semi-straight (Fig. 2e). Conversely, the cell walls of both *Frullania rostrata* and *Frullania pseudomeyeniana* areflexuose with indistinct trigones, and with small, nodulose intermediate thickenings throughout the lobule, from the base to the apex (Fig. 2f).

**Figure 3. F3:**
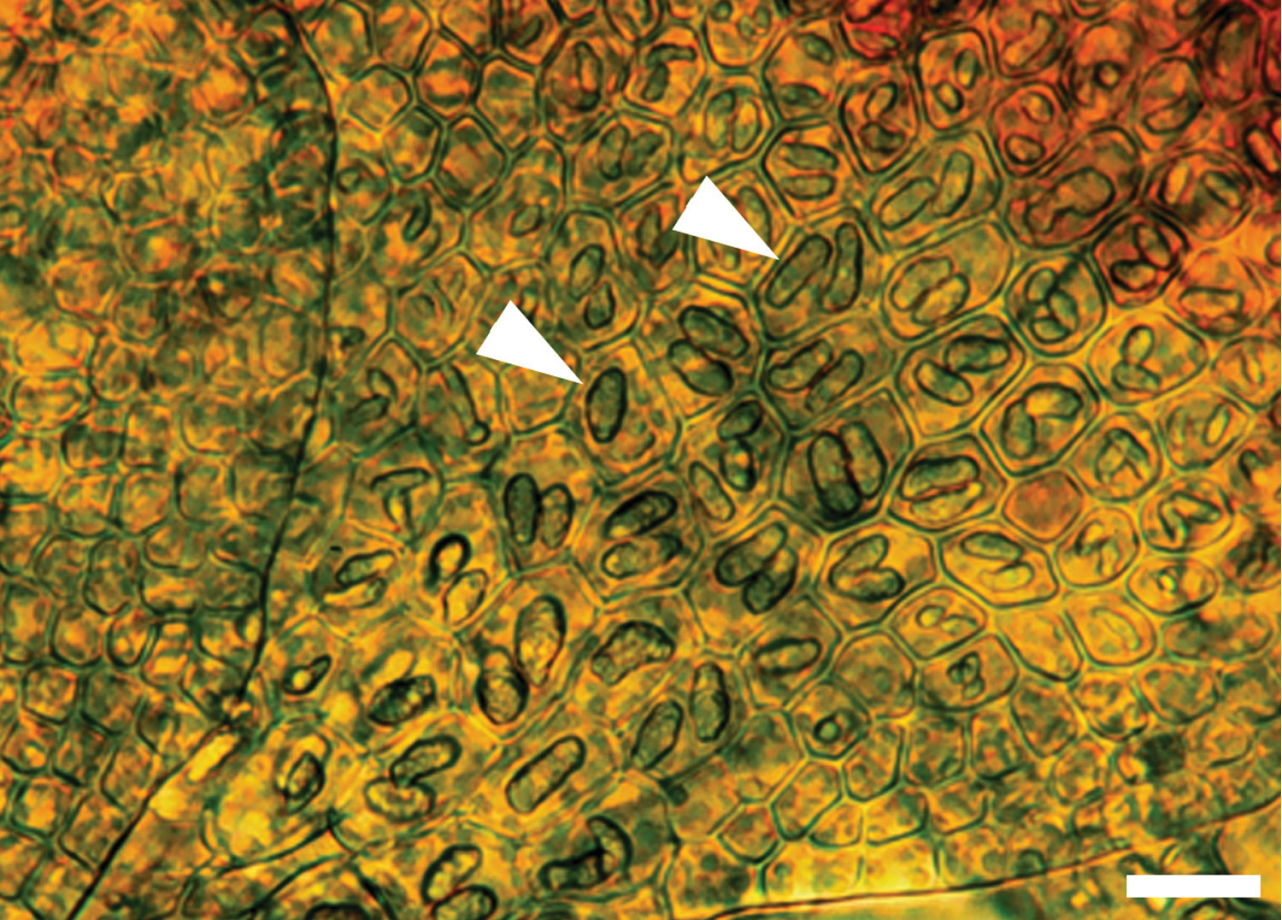
Oil bodies in a leaf shoot of *Frullania knightbridgei* illustrating, in region of arrow, the 1–2 large oil bodies per cell. Scale bar = 20 µm.

The unique cell anatomy of the leaf lobe in *Frullania knightbridgei* furtherplaces it into an isolated position within subg. *Microfrullania*; this species is seemingly unique in having a group of conspicuously enlarged cells, originating from the base of the lobe and extending 10–14 cells out toward the apex, forming a partial band or pseudo vitta up to 4–6 cells wide (Fig. 3). The cells are enlarged to accommodate the typically 2 large oil bodies. The features of the oil bodies are unique within *Frullania* subg. *Microfrullania*. In those species examined thus far, the oil bodies of the leaf lobe median cells number from 2–4(5) per cell, are of small size and lack any significant ornamentation, almost appearing as homogeneous oil droplets (von Konrat et al. 2006a, 2010) (Fig. 2c–d).

The position of the lobules in relation to the stem as well as styli shape and form are often used to help distinguish between taxonomic units of varying levels in *Frullania* taxonomy. Lobule position varies in *Frullania knightbridgei*, ranging from parallel to subparallel with the stem or with the lobule spreading at a strong angle, so that the lobuli are tilted inwards. *Frullania pseudomeyeniana* and some phenotypes of *Frullania rostrata* also have lobuli that lie almost parallel or subparallel to the stem for both species. *Frullania magellanica* also has at least some phases with lobules more or less parallel to the stem (Engel 1978). Interestingly, the parallel lobule position is typically a feature associated with species of subg. *Thyopsiella*. Thus lobule position must be used secondary to and in collaboration with more salient characters in circumscribing *Frullania* subg. *Microfrullania*.

Historically, characters associated with the capsule wall and spore surface ultrastructure have rarely been utilized in *Frullania* systematics (von Konrat et al. 2006b). Yet, it is clear that characters associated with these structures have great utility at various taxonomic levels (eg., von Konrat et al. 1999, 2006b, 2010). The spores of *Frullania knightbridgei* have a “rosette” with conspicuous protuberances lacking secondary branches and deposits – a feature used to help characterize *Frullania* subg. *Microfrullania* (Hentschel et al. 2009). The spores can also be used to distinguish *Frullania knightbridgei* and *Frullania rostrata* (Table 3, Fig. 5). Differences are also reflected in the epidermal wall of the capsule. In *Frullania knightbridgei*, the walls are nodular, where the lobes extend irregularly for a short distance over the tangential face toward the centre of the cell and have intermediate thickenings near the middle of the longer walls (Fig. 5a). In *Frullania rostrata*, the lobes extend toward the centre of the tangential face for a short distance, as short rounded or obtuse lobes and the juxtaposed corner thickenings form 3–4 lobed figures; intermediate thickenings are also lacking (Fig. 5b).

**Table 3.** Initial branching appendages.

**Branching**	***Frullania knightbridgei***	***Frullania truncatistyla***	***Frullania rostrata***
Branching type	*Frullania*-type	Usually *Frullania*-type, occasionally *Lejeunea*-type	Usually *Frullania*-type, occasionally *Lejeunea*-type
First branch underleaf (BUL1)	1 ventral, explanate, bilobed segment + 1 dorsal saccate segment	1 ventral, explanate, bilobed segment + 1 dorsal saccate segment	1 ventral, explanate, bilobed segment + 1 dorsal saccate segment
First branch leaf (BL1) initial appendages	± characteristic of normal stem leaves	Variable, either elobulate, and explanate to sulcate, or ± characteristic of normal stem leaves	Variable, either elobulate, and explanate to sulcate, or ± characteristic of normal stem leaves

**Figure 4. F4:**
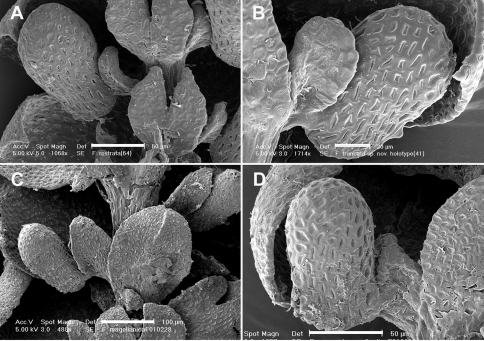
Lobule position and styli. **A** *Frullania rostrata* **B** *Frullania truncatistyla* **C** *Frullania magellanica* **D** *Frullania knightbridgei*Scale barsA, D = 50 µm; B = 20 µm; C = 100 µm.

**Figure 5. F5:**
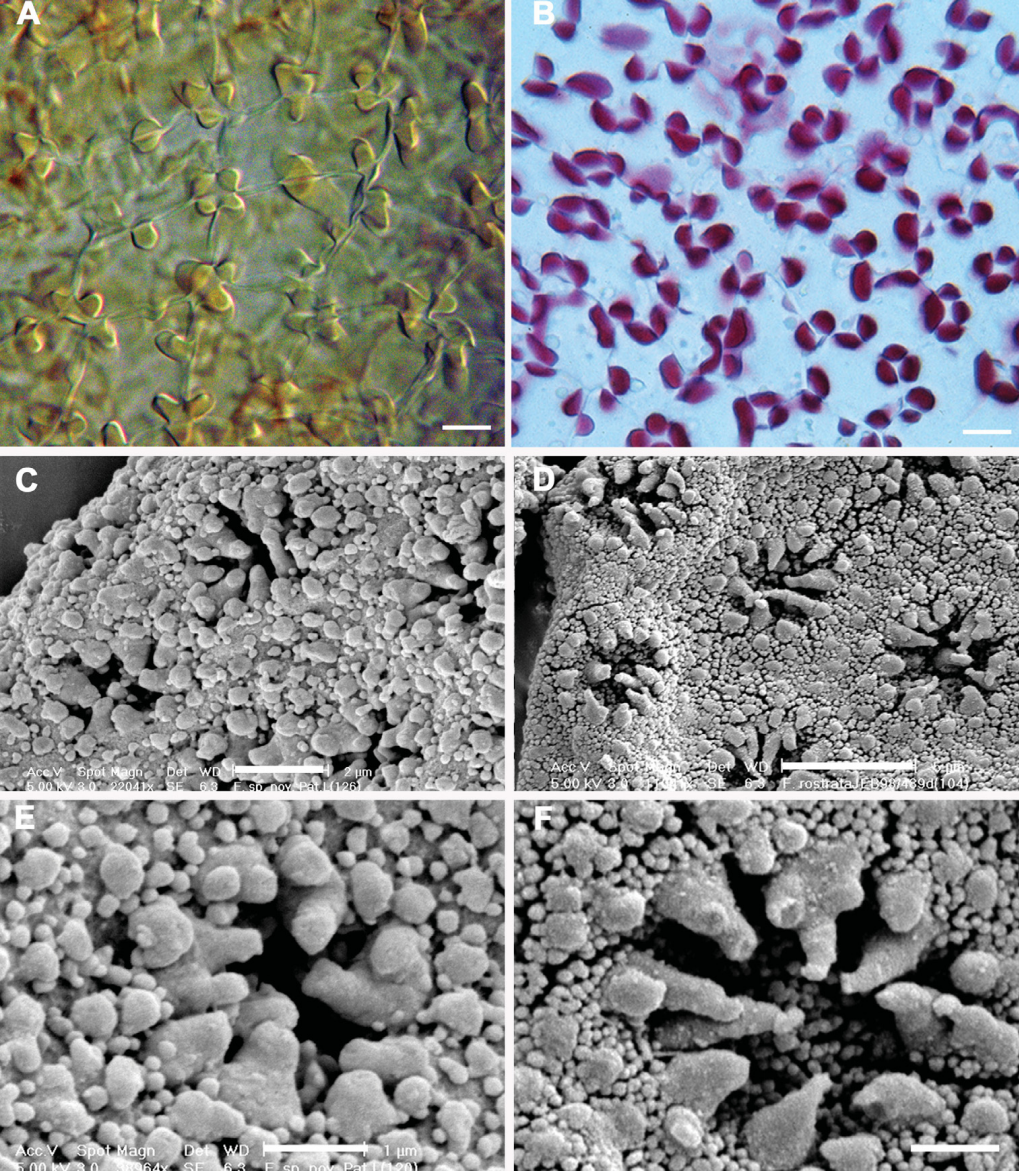
Epidermal layer of the capsule wall and spore surface ultrastructure. **A** Epidermal layer of *Frullania knightbridgei***B** Epidermal layer of *Frullania rostrata***C, E** Spore surface of *Frullania knightbridgei***D, F** Spore surface of *Frullania rostrata*.Scale bars A, B = 10 µm; C = 2 µm; D = 5 µm; E,F = 1 µm.

Tables 3–5 summarizes the characters differentiating *Frullania knightbridgei* from two morphologically similar species that it might be confused within the New Zealand botanical region - *Frullania truncatistyla* and *Frullania rostrata*. This includes critical characters associated with initial branching appendages (Table 3), oil bodies and cell anatomy (Table 5, Fig. 2, 3), stem anatomy, leaf lobe, underleaf, leaf lobule and stylus (Table 4, Fig. 4), perianth, and sporophyte, including spores (Table 4, Fig. 5).

**Table 4.** A comparison of morphological characters between three New Zealand species, *Frullania knightbridgei*, *Frullania truncatistyla* and *Frullania rostrata*.

**Character**	***Frullania knightbridgei***	***Frullania truncatistyla***	***Frullania rostrata***
Shoot width	To µm 1000 µm	To 550 µm	To 1100 µm
**Stem**			
Cortical cells	10–34	7–12	10–25
Medullary cells	12–28	8–14	12–30
**Leaf-lobe**			
Apex	Rounded	Acute	Rounded to sharply acute
Median cell size	Dimorphic; central band of cells similar to basal cells	Markedly uniform and smaller than basal cells	Markedly uniform and smaller than basal cells
**Leaf-lobule**			
Position in relation to stem	Often almost parallel with the stem, or at most lobules at angles of up to ca. 25°	Angles of 30–50 (60)u with the stem so that lobules tilted outwards	20) 30–50 (60)° with the stem so that lobules tilted outwards
Length:width ratio	1.75–2:1	1.0–2.0:1	1.5–2.25:1
No. of cells in<br/> circumference	To 25	To 20	To 28
Colour	Bicoloured	Uniform colour similar to other organs	Uniform colour similar to other organs
Cell walls toward lobule apex	Semi straight	Flexuose	Flexuose
**Stem-underleaf**			
Margin	Entire	Entire	Entire; occ. angulated or toothed.
No. of cells wide (lobe)			
Width in comparison to stem	(2) 2.5–3.5 (4)	1.0–1.25	(1) 2–3.5 (5)
**Stylus**			
Shape	± triangular	Subrectangular, apex ± truncate to subtruncate	Variable, subtriangular to foliaceous to sickle-shaped
Sexuality	Dioecious	Dioecious	Dioecious
Archegonia No.	1	1	1–2
Perianth	Plicate, to 6-keeled	3-keeled	3-5 keeled
**Spores**			
Form of projections comprising rosette	Taper gradually to a rounded or truncate apex	Not seen	Taper gradually to a rounded or subacute, often hooked apex

**Table 5.** Characters associated with oil bodies of the leaf-lobe.

**Character**	**i*Frullania knightbridge***		**a*Frullania truncatistyl***	**a*Frullania rostrat***
**General**	**Dimorphic**		**Monomorphic**	**Monomorphic**
	Type 1	Type 2		
**Median cells**				
Size	(2) 3–7 (9) µm in diam. to (5) 6–11 (13) × (4) 5–10 (12) µm	(1) 2–4 (5) µm in diam. to (2) 3–5 (6) µm × (1) 2–3 (4) µm	(1) 2–3 (4) µm to (2) 2–3 (5) 3 (1) 1–2 (3) µm	(1) 2–3 (4) µm in diam. to (2) 3–5 (6) µm × (1) 2–3 (4) µm
Shape	Rarely subspherical usually ovoid or ellipsoidal	Spherical to ovoid or ellipsoidal	Spherical to ovoid or ellipsoidal	Spherical to ovoid or ellipsoidal
Number	1-2	2–3 (4)	2–3 (4)	2–3 (5)
Density (collectively)	Occupying almost entire cell lumen	Occupying <25% of cell lumen	Occupying <25% of cell lumen	Occupying <25% of cell lumen
Surface	Appearing granular	Appearing ± homogeneous	Appearing ± homogeneous	Appearing ± homogeneous
**Basal cells**				
Number	1–2		3–5	(2) 3–5 (6)

## Taxonomic treatment

Artificial key distinguishing *Frullania knightbridgei* from morphologically allied species of subg. *Microfrullania*, including those distributed in New Zealand.

Fertile and sterile features combined

**Table:** 

1	Leaf-lobes with denticulate to coarsely dentate margins	Sect. *Microfrullania*: *Frullania chevalieri*, *Frullania microscopica*, *Frullania parhamii* [incl. New Zealand, New Caledonia, and Fiji]
–	Leaf-lobes with entire margins, lacking any form of marginal dentition	2
2	Stylus a distinct, obovate to subrectangular in shape with a truncate apex; a distinctive angular projection on the lobule immediately above the slit; plants small (c. 250–500 µm)	*Frullania truncatistyla*[North Island, South Island, Stewart Island]
–	Styli sickle-shaped, subtriangular to triangular; distinctive angular projection absent (not to be confused with the ± discoloured, gibbous, cell above mouth); plants small to medium	3
3	Dioecious, gynoecia terminal on leading stems with subfloral systems (i.e., subfloral innovations or subfloral branches); stylus small to medium, 0.25–0.5× the length of the lobule), typically stylus with up to 10–35 cells	4
–	Monoecious, gynoecia on short lateral branches lacking subfloral systems; stylus typically large, 0.75–1.0× the length of the lobule, stylus with up to 100 cells total	*Frullania magellanica* [Chile]
4	Lobules typically at an angle in relation to the stem, leaf-lobe median cells smaller than basal cells and with 3–4 oil bodies per cell, occupying <50% of the area of the cell lumen; perianth typically 3-keeled	*Frullania rostrata* s. l. [Australasia]
–	Lobuli varied, typically subparallel to the stem; a band (pseudovitta) of median cells (of leaf-lobe) as large as basal cells and with 1–2 oil bodies per cell, occupying almost entire cell lumen (where known); perianth plicate 5–6 keeled	5
5	Leaf lobes often squarrose;main stem underleaves small, ± as wide as stem;leaf lobuleclavate,cell walls distinctly flexuose toward the lobule apex	*Frullania pseudomeyeniana* [New Caledonia]
–	Leaf lobes flat, not squarrose;main stem underleaves medium to large, wider than stem;leaf lobulecylindrically helmet-shaped, cell walls becoming distinctly semi-straight toward lobule apex	*Frullania knightbridgei* [Stewart Island, Auckland Island]

### Frullania knightbridgei

von Konrat & de Lange sp. nov.

http://species-id.net/wiki/Frullania_knightbridgei

Figs 2 [Fig F3] [Fig F4] [Fig F5] [Fig F6] –7 

#### Diagnosis.

*Frullania knightbridgei* is similar to *Frullania rostrata* (Hook.f. & Taylor) Hook.f. & Taylor ex Gottsche et al., but differing by the presence of large oil bodies that occupy almost the entire lumen of the basal and median cells of the leaf lobe, and the often bicoloured lobules, which usually lie almost parallel to the stem. The leaf lobule cell walls of *Frullania knightbridgei* are distinctly semi-straight toward the lobule apex whereas in *Frullania rostrata* the cell walls are distinctly flexuose toward the lobule apex.

#### Type.

New Zealand: Stewart Island: Rakiura/Stewart Island National Park, 500 m. from North Arm Hut, on bark of *Dracophyllum longifolium* overhanging water on margin of Patterson Inlet, canopy of stunted *Dacrydium cupressinum* and *Metrosideros umbellata*. Near sea level, 46°52'55"S, 168°01'04"E, 12 Dec 1999, *Metrosideros von Konrat 99/12-09* (holotype AK; isotypes CHR, F).

#### Description.

Plants small to medium (main shoots to 600 µm wide), forming olive-green, copper-brown, to black patches, closely to loosely adhering to substrate. Leading stem 15–25 mm long and to 90µm in diameter, 6–9 cells wide, little differentiation between cortical cells (18–24 in no.) and medullary cells (14–28 in no.), the former often slightly smaller than the latter, both with firm walls, lumen irregularly shaped. Branching often regularly pinnate, occasionally bipinnate to rarely tripinnate, branches with progressively smaller leaves; *Frullania*-type branching. First branch underleaf (BUL1) always with three distinct segments, the ventral lamina divided for ^1^/_3_–^2^/_3_ its length into two unequally or subequally sized lobes + 1 dorsal saccate lobe; First branch leaf (BL1)usually± characteristic of normal stem leaves (i.e. 1 explanate dorsal lobe + 1 saccate lobule + 1 stylus). Stemleavesof main branchflat when dry and wet, slightly imbricate to contiguous, suborbicular to broadly ovate, to 375 µm long × 350 µm wide with incurved distal margins, dorsal margins extending beyond the farther edge of the stem, rounded apices, non-auriculate and ± subtruncate at the base, entire margins, smooth dorsal surface. Lobules remote from the stem (lobule attached to stem by 3–4 cells) and usually almost parallel with the stem so that the long axis of the lobule is ± parallel with the main stem (or at most lobules at angles of up to ca. 25° with the stem so that lobules only very slightly tilted outwards); lobules often bicoloured with the basal 2–5 cells towards the mouth (up to 0.25 of the lobule) hyaline to subhyaline, in contrast to the olive-green to brown pigmentation elsewhere; cylindrically helmet-shaped (orbicular in cross-section with up to 25 cells in circumference); lobules ± medium (lobule area obscuring no more than 0.25 × the exposed area of the dorsal lobe), ca. 1.75–2 × long as wide, 110–200 µm long × 60–100 µm wide (up to 12–14 cells high × 6–8 cells wide); ± equally inflated throughout (so that the sides of the lobule are ± parallel), the opening wide, extending only slightly along the abaxial lobule margin; ca. ^2^/_3_ from lobule apex there is usually a ± discoloured, gibbous, cell (the free margin of the cell with a heavily thickened wall); mouth nearest the stylus, truncate at base then cells with septa between adjacent cells ± swollen, the mouth thus then becoming crenulate-sinuate; lobule usually hyaline near mouth, lobule apex obtuse, surface of lobule smooth. Stylus medium in size (^1^/_3_–^2^/_3_× the length of the lobule), ± triangular, up to 60 µm long × 50 µm wide, (4) 5–6 (7) cells wide at base, (10) 12–24 (30) cells in total, occasionally with a poorly developed slime papilla at the apex.Underleaves of leading stems bilobed, obovate to rotundate, at most only contiguous with lobules, underleaves contiguous to distant from each other, usually long as wide, occasionally slightly longer than wide, (2) 2.5–3.5 (4)× the stem in width, to 100–175 µm long × 100–150 µm wide, broadest at middle, free lateral margins always entire; apex of underleaf bilobed to ^1^/_3_–½ its length, lobes separated by a V-shaped sinus, the lobes 9–14 cells wide at base and with blunt to subacute or rounded apices. Rhizoid-initial area present near base of underleaf, rhizoids often seen, subhyaline, in bundles, to 400 µm long. Not strictly microphyllous, lobules of secondary stems ± similar size to main stem, but lobes and underleaves of secondary branches slightly smaller than those of leading stems.

Leaf-lobe: to 20 cells long, from base to apex, by 35 cells at widest region; with a band of conspicuously enlarged cells originating from the lobe base and extending out towards the lobe apex 10–12 cells, and up to 6 cells wide at the widest region. Lobe marginal cells ± rectangular to subquadrate, small to 8 µm long × 6 µm wide, hyaline walls subequally thickened, cell cavities brownish red. Cells of the middle region of the lobe are ± dimorphic in size; Type One [see below]: 4–6 rows of median cells, cells to 30 µm long × 22.5 µm wide (usually 2–2.75 × long as wide), thus similar in size to basal cells; Type Two [see below]: cells gradually becoming reduced in size (median cells to 15 µm long × 10 µm wide, usually 1.25–2 × long as wide, between central band of enlarged cells and lobe margin). Both cell types usually pentagonal or hexagonal, hyaline walls subequally thickened, intermediate thickening rare to absent, wall thickness to 2.75 µm wide, cell cavities of median cells brownish red. Cells becoming gradually larger basally, cavities of the basal cells to 40 µm long × 25 µm wide; walls of basal cells with small indistinct trigones and semi-straight walls without any intermediate thickenings, walls and cavities brownish red. Median cells of underleaves vary in shape and size, cells with heavily equally-thickened walls so that the hyaline trigones and intermediate thickenings become indistinct. Median cells oflobule as long as wide or slightly longer than wide, cell cavities to 14 µm long × 9 µm wide; cells near lobule mouth, irregular in shape with flexuose walls, indistinct trigones and occasional small nodulose intermediate thickenings; towards the lobule apex, cells gradually becoming more regular in shape, quadrate to rectangular and the cell walls becoming semi-straight.

Oil bodies of lobe median cells dimorphic. Type One: (1)2(3) per cell, very large, occasionally spherical (2) 3–7 (9) µm in diam. but usually ovoid or ellipsoidal (5) 6–11 (13) × (4) 5–10 (12) µm, finely granular, occurs from basal cells through to central region of the lobe, occupying 3/4 to almost the entire cell lumen. Type One oil bodies larger than chloroplasts (if chloroplasts present at all). Type Two: 2–3 (4) oil bodies per cell, typically small, spherical (1) 2–4 (5) µm in diam. to ovoid or ellipsoidal (2) 3–5 (6) µm × (1) 2–3 (4) µm, subhyaline, without any significant, visible, internal structure i.e. giving the appearance of being almost homogeneous; these oil bodies often similar in size or slightly smaller than chloroplasts, occasionally slightly larger than chloroplasts. Oil bodies of lobule and underleaf of Type One. Asexual reproductionnot recognized.

Plants dioecious, male plants slightly smaller than female plants. Androecia subspherical to spicate, 2–4 (6) pairs of bracts, terminal, usually on very short-stalked branches arising from the main stem, or occasionally from secondary branches (stalk with (1) 2–3 (4) vegetative leaf lobes). Gynoecia terminal on main or leading stem often bearing a subfloral innovation arising 3–4 bract-pair cycles back from the perianth or gynoecia. Innermost bract unequally bilobed; bract-lobe, lobule and innermost bracteole all with entire margins. Bract-lobe ovate to oblong, narrowed toward the rounded or subacute-acute apex, bract-lobule ovate–lanceolate, subacute; innermost bracteole free from bracts, oblong-ovate to oblong-obovate, ca. ^1^/_2 _bilobed, lobes convex-sided, subacute at apex, entire margins. Marginal cells of bract and bracteole ± subequally thickened, but towards the median cells, trigones becoming large and bulging. One archegonium per gynoecium. Perianth freely emergent, 900 µm long × 500 µm wide, perianth plicate, 1–2 dorsal keels + 2 lateral keels + 1–2 ventral keels, smooth surface, oblong-ovate, tapering towards the apex into a short beak; perianth beak cylindrical, with a smooth mouth but the inner beak surface densely covered with large single-celled protuberances.

Walls of epidermal layer of capsule wall nodular where the lobes extend irregularly for a short distance over the tangential face toward the centre of the cell and have intermediate thickenings near the middle of the longer walls. Spores globose, 35–45 µm at widest axis, spore wall papillae densely distributed, equatorial face interspersed with 8–10 rosettes, 2.5–3 µm wide in the equatorial diameter, bearing a ring of 7–10 conspicuous primary projections, 0.75–1.5 µm long × 0.5–1.0 µm wide at base (often with a 1.5–2:1 length to width ratio), tapering gradually to a rounded apex, never papillate or bearing secondary short branches.

**Figure 6. F6:**
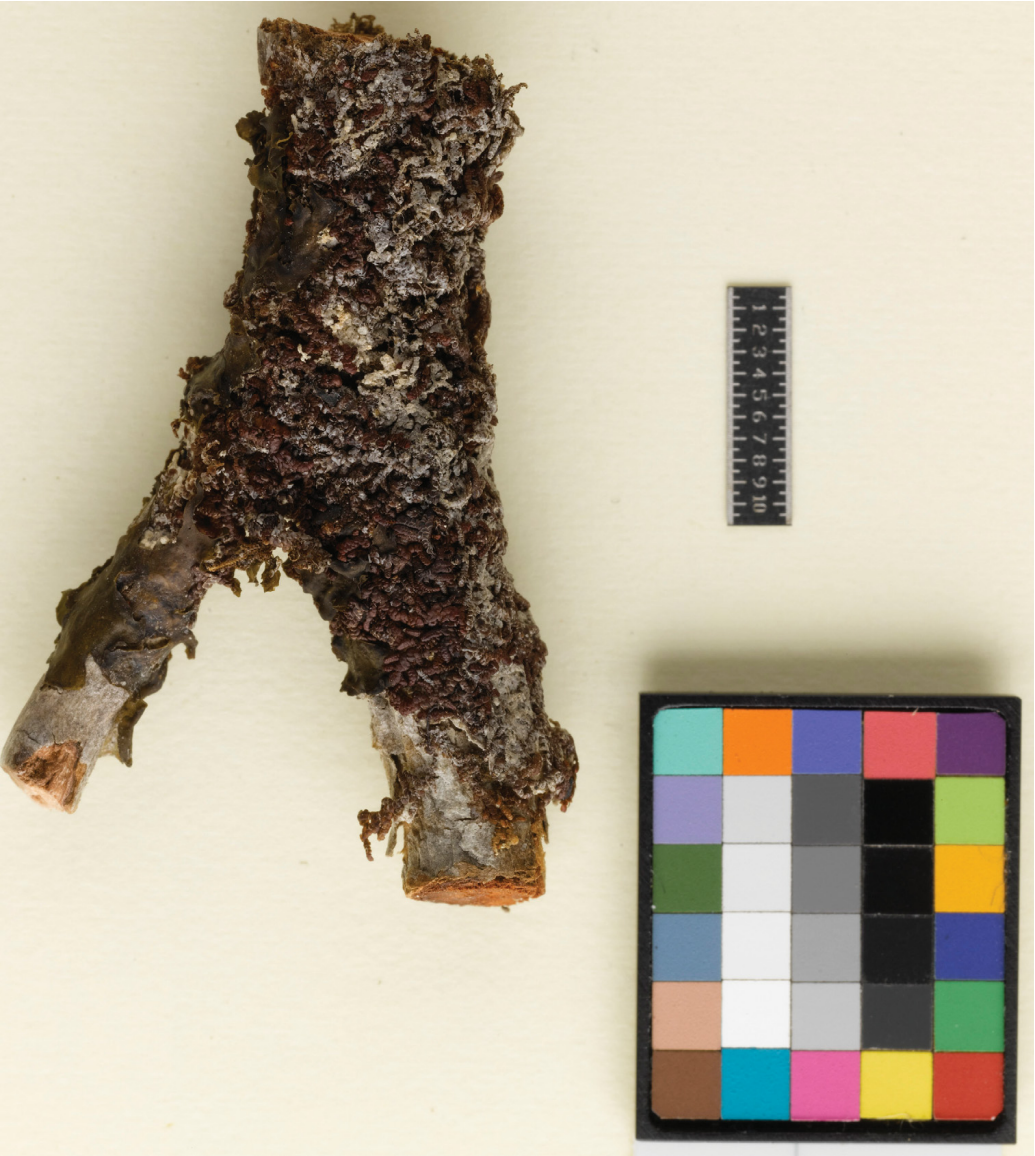
*Frullania knightbridgei* on twig, Auckland Island. (Coll. *CommonC893B*).

**Figure 7.**
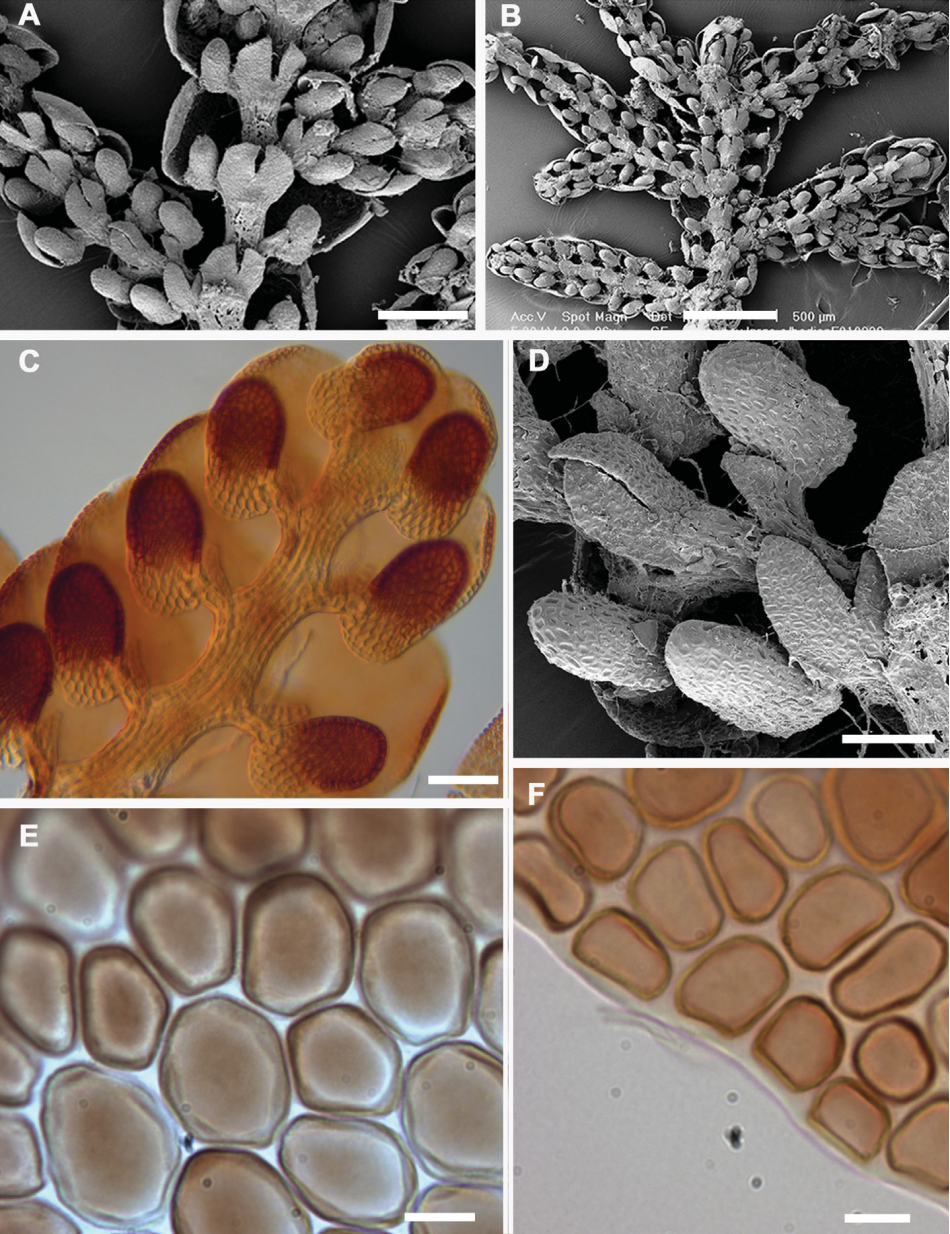
*Frullania knightbridgei* **A, B** Main stem, ventral view. **A** Main stem and lateral branches, lobules subparallel in relation to the stem and occupying ca. 25% of the exposed surface of the dorsal lobe **B** iIllustrating terminal position of the gynoecium with 2 subfloral branches immediately below **C** Bicoloured leaf-lobules **D** Initial branching appendages **E** Median cells of the leaf-lobe with subequally thickened cell walls **F** Basal cells of the leaf-lobe. Scale bars A = 200 µm; B = 500 µm; C,D = 50 µm; E, F = 10 µm.

#### Etymology.

The species epithet *knightbridgei* is named in honour and memory of an esteemed New Zealand conservation botanist and ecologist, Phil (Philip) Ian Knightbridge (1969–2011) who passed away in April 2011. This southern species of Aotearoa-New Zealand is a small tribute to Phil by the senior authors who knew him as a colleague and friend.

#### Distribution and ecology.

This species is currently known from only four collections; two from the shore margin of Paterson Inlet, Stewart Island and two from the Auckland Islands. *Frullania knightbridgei* probably has a more extensive distribution than is currently known and it is quite likely that it resides unrecognized in New Zealand herbaria filed as a form of *Frullania rostrata*. Nevertheless, it would appear that *Frullania knightbridgei* is a species of southern distribution and it would be interesting to see if the distribution extends south to the Campbell Islands of the New Zealand botanical region; further field work is required to establish if the species grows on the other two main islands of the New Zealand archipelago, North and South Islands, it should for example be looked for along the Fiordland and Foveaux Strait coastline of the southern South Island. Note the type of *Frullania pseudomeyeniana* is a high elevation taxon at  1,100 m whereas the New Zealand taxon is seemingly restricted to or near the shoreline or low elevation.

*Frullania knightbridgei* is noteworthy in comparison with the majority of *Frullania* species in New Zealand for it appears to be a salt tolerant species. This is clearly evident in Stewart Island/Rakiura where *Frullania knightbridgei* was growing on twigs of *Dracophyllum* immediately adjacent the shoreline, at a height of about 50 cm above the sea. It was evident that for at least some periods of the year, this represented a harsh coastal environment where significant exposure to salt spray from violent wave action would be common.Elsewhere, *Frullania ericoides* reportedly develops in rock crevices exposed to sea in the Madeiran archipelago (Sim-Sim and Sergio 1992), and Schuster (1992) reported a variety of *Frullania kunzei* growing in mature mangrove swamp forest where salt spray and even rare submersion was possible. The high rainfall of the region, providing fresh water, is possibly a critical factor as Engel and Schuster (1973) described for tidal zone liverworts in southern Chile. Interestingly however, Engel and Schuster (1973) noted for Stewart Island a notable “lack of any Hepaticae in the intertidal zone where sea spray is a factor”. At least in the New Zealand botanical region, it is clear this is a habitat area that has traditionally been underexplored for liverworts. It would also be interesting to perform glasshouse experiments to investigate test the extent of salt tolerance in these organisms.

#### Paratypes:

Stewart Island: Rakiura/Stewart Island National Park, 500 m. from North Arm Hut, on bark of *Dracophyllum longifolium* var. *longifolium* overhanging water on margin of Patterson Inlet, canopy of stunted *Dacrydium cupressinum* and *Metrosideros umbellata*, 12 Dec 1999, *M. von Konrat 99/12-10* (AK, F); Auckland Island: Open *Oreobolus* cushion and *Chionocloa* tussock with scattered groves of *Metrosideros*, above Ranui Cove, 50°32'12"S, 166°16'24"E, 11 Dec 1972, *R. Common C893B* (CHR, F).

## Supplementary Material

XML Treatment for Frullania knightbridgei
